# Botulinum Neurotoxin Treatment of Camptocormia- A Critical Review and Update

**DOI:** 10.5334/tohm.1230

**Published:** 2026-08-05

**Authors:** Ava G. Tohidian, Ali Ghaseminejad-Bandpey, Bahman Jabbari

**Affiliations:** 1 Royal college of Surgery, Dublin, IE; 2 Glenn Biggs Institute for Alzheimer’s and Neurodegenerative Diseases, University of Texas Health Science Center at San Antonio, San Antonio, TX, US; 3 Yale University, US

**Keywords:** camptocormia, botulinum neurotoxin, onabotulinumtoxinA, rectus abdominis, external oblique, Parkinson’s disease, dystonia

## Abstract

Camptocormia is a disabling postural disorder characterized by involuntary forward flexion of the thoracolumbar spine that resolves in the supine position. It is mostly associated with Parkinson’s disease and parkinsonian syndromes, but may also occur in dystonia, paraspinal myopathies, and after exposure to some drugs. Available treatments, including postural aids, medication adjustment, and deep brain stimulation, are often only partly effective and may carry risk. Because overactivity of trunk flexor muscles is thought to contribute to camptocormia, botulinum neurotoxin (BoNT) has emerged as a potential treatment. This review critically evaluates the published literature on BoNT treatment of camptocormia. We searched Medline, Google Scholar, and Scopus for English-language publications published through April 1, 2026, identifying nine reports including three case series and six single case reports. No randomized placebo-controlled trials were found. Most studies used onabotulinumtoxinA, but injection targets, dosing, and outcome measures varied. Improvement in posture, reduced pain and functional benefits were described. The most consistent benefit was with bilateral injection of the rectus abdominis and external oblique muscles, whereas isolated psoas or iliopsoas injections were less effective. Overall, BoNT appears promising for the treatment of camptocormia; however, randomized, placebo-controlled trials are needed to confirm efficacy and establish optimal treatment protocols. The studies should be conducted with caution because long-term outcome data are limited and reported efficacy has been modest.

## Introduction

Camptocormia (CC), derived from Greek terms kamptos (bent) and kormos (trunk), is characterized by bent posture of the thoracolumbar spine which disappears in supine position. It was coined by Souques and Rosanoff Saloff (camptocormie) describing the posture of bent trunk in some soldiers participating in World War I [1]. They considered the soldier’s CC to be psychogenic. Over the years and in the civilian population, it became apparent that most cases of camptocormia are not psychogenic but are associated with different disease conditions such as Parkinsonian and dystonic disorders (Parkinson disease, multiple system atrophy, different dystonias) [2], as well as systemic or paraspinal myositis [3] and exposure to certain drugs.

In the sizeable reported series of patients with CC, the reported frequency of different causes depends on the type of the clinic where the reports had been generated [4]. One group of academic neurologists have found that 68.8% of 16 studied patients with CC had Parkinson’s disease (PD) [5], whereas in a report from rheumatologists, only 8 of 63 patients (12.7%) had PD while most of the rest, had different myopathic conditions with or without inflammation [6]. Other notable causes of camptocormia are tardive dyskinesia, use of psychotropic or dopaminergic drugs as well as opioids [7].

Mechanistically, CC is caused by imbalance between the function of spine extensors (paraspinal muscles) and spine flexors with overactivity of flexors leading to a bent trunk. The major trunk flexors are rectus abdominis (RA) and external oblique (EO) muscles as well as iliopsoas (IS). In case of external oblique, unilateral contraction turns the trunk to left or right while bilateral contraction bends it. Rectus abdominis and EO receive their innervation from T7-T12 segments of the spinal cord. The innervation from T7-T11 is supplied through thoracoabdominal nerves - a continuation of intercostal nerves, whereas, innervation from T12 segment comes from the subcostal nerve. Ilopsoas muscle is innervated by L1 to L3 and sometimes by additional L4 nerve roots.

Clinically, severe bent thorax interferes with walking and makes the patients prone to fall. Moreover, prolonged CC can also cause chronic back pain. The abnormal bent position, resolves in supine position or rising the hands when the patient facing a wall pretending to climb it. However, the abnormal posture returns again shortly upon standing.

Treatment of camptocormia is difficult and current modes of therapy are either partially effective or carry the risk of unpleasant side effects. In some patients, wearing a plaster corset or a 6Kg back pack, as well as applying high frequency waves (HFW) to stimulate spine extenders have been reported to be helpful [8,9,10]. When camptocormia is associated with Parkinson’s disease (the most common movement disorder associated with camptocormia), increasing dopaminergic medications may help some patients.

The preferred surgical approach in camptocormia is deep brain stimulation (DBS) of subthalamic nucleus or globus pallidus. A recent meta-analysis of 20 studies concluded that DBS is an effective treatment of camptocormia in patients with Parkinson disease [11]. However, though uncommon, local brain infection or bleed, as well as mechanical issues such as malfunctioning DBS unit or breaking unit wires, makes many patients hesitant to accept this treatment.

Efficacy of botulinum toxin injections to improve dystonia and abnormally increased muscle tone [12,13] has encouraged researchers to study the role of botulinum toxin therapy in camptocormia. This review provides an update of published literature on this subject and critically assesses the validity of applied techniques for improving camptocormia.

## Research Design

We searched Medline, Google Scholar and Scopus for clinical trials, case series, case reports and book chapters published up to April 1^st^, 2026 using the search terms of botulinum neurotoxin, botulinum toxin and camptocormia. Two of the authors (AT and BJ) searched the literature independently while one author (AGB) subsequently reviewed and verified the search results. Included in the search, were clinical trials, case series, case reports and information from book chapters. Excluded from this search, were manuscripts published in language(s) other than English, abstracts and review articles. The novelty of this review is that it critically reviews the issue of injection technique and toxin dose in CC and provides the latest data in the literature on this subject.

## Results

Our search disclosed 9 reports on treatment of camptocormia with botulinum neurotoxin. Three of nine were small series of 4 to 10 patients; the other 6 were single case reports (Table 1). There was no randomized blinded, placebo- controlled study. Seven studies used onabotulinumtoxinA (Botox) injections, one study applied abobotulinumtoxinA (Dysport) and another employed incobotulinumtoxinA. Five of 9 studies used standard rating scales. Two of nine studies reported side effects (Table 1).

**Table 1:** Summary of finding in the reported manuscripts on treatment of camptocormia with botulinum neurotoxins.


AUTHORS AND DATE	#PTS	MUSCLE(S) INJECTED	TYPE OF TOXIN	DOSE IN UNITS	ASSESSMENT	RESULTS	SIDE EFFECTS

Azher & Jankovic, 2005 [5]	9	Rectus abdominis (bilateral)	onaA	300–600 per treatment visit	Clinical: posture (degree of trunk flexion); Functional improvement; UPDRS (for PD pts); Video documentation	4 patients showed moderate to marked improvement (lasting 8–16 weeks), while remaining 5 patients had no significant improvement	Not mentioned

Von Coelln et al, 2008 [14]	4	Psoas major: Two patients bilateral. Two patients unilateral	aboA	Initial: 500 per side, Maximum: 1,500 per side	Clinical exam; Patient subjective reporting; Objective posture measure (height)	Temporary improvement with functional gain (1 patient); Worsening of posture and general condition (1 patient). Overall, no significant benefit.	Mild hip flexor weakness and transient local itching at injection site (1 patient), mild transient functional impairment (1 patient)

Colosimo and Salvatori, 2009 [15]	2	Ileopsoas (bilateral), rectus abdominis (bilateral)	onaA	Iliopsoas: 300; rectus abdominis: 200	Clinical: objective and subjective assessment of posture	No objective or subjective improvement.	Not mentioned

Fietzek et al, 2009 [16]	10	Rectus abdominis (bilateral), Iliopsoas (bilateral)	Inco-A	100–300 U	UPDRS, Times Up & Go, Goal Attainment Scaling	No significant improvement	Mild muscle soreness at injection site (2 patients)

Wijemanne et al, 2014 [17]	1	Initially: Rectus abdominis (bilateral)Subsequently: External oblique (L) and Rectus abdominis	onaA	Initially, 200 into each rectus with additional injection of 200 into each external oblique two months later	Degree of trunk flexion; ability to lie flat; functional ability; MDS-UPDRS III scores (ON vs OFF); EMG; CT (to assess muscle anatomy and physiology)	Limited improvement initially but marked improvement following second treatment with additional external oblique injection. Benefits were sustained over 18 months with repeated injections.	None mentioned

Matsumoto et al, 2018 [18]	1	Right lumbar paraspinal muscles (L2-L4)	onaA	Initial: 20 Repeat injection one month later: 30	Posture and pain; posture X-ray (before and after); CT (assessed muscle volume; EMG	camptocormia and Pisa syndrome improved with reduction in abdominal pain.	None

Todo et al, 2018 [19]	6	External oblique (bilateral)	onaA	75–90 per side; 150–180 per patient	CC angle; Subjective symptom relief, Pain (VAS), Functional measures	Significant improvement of CC angle in all pts (Median 38° → 18°), 4 of 6 patients reported pain relief and increased gait speed and stride	None

Jabbari, 2022 [20]	3	Rectus abdominis and external oblique bilaterally	onaA	200 per side into rectus abdominis; 150 per side into external oblique	Abdominal Pain measured by VAS; Improvement of posture measured by CC angle, treatment combined with physiotherapy	Significant improvement of CC angle in all 3; Significant pain improvement in one: VAS score dropped from 8 to 2	None

De Monte et al, 2026 [21]	1	Rectus abdominis (bilateral) External oblique (bilateral)	onaA	Rectus abdominis: 30 per side; External oblique: 50 per side	UMSARS Part II; Timed Up and Go; 10-Meter Walk Test; Wearable sensor gait analysis; EMG; Static posturography	Posture score, walking velocity, and step length all improved. BoNT treatment was superior to spinal stimulation.	None


CC: Camptocormia; OnaA: onabutulinumtoxinA (Botox); aboA: abobotulinumtoxinA (dysport); GAS: goal attainment scaling; VAS: Visual Analogue Scale; UPDRS: Unified Parkinson Disease Rating Scale; UMSRS: Unified Multiple System Atrophy Rating Scale.

## Discussion

Successful botulinum toxin treatment of any medical condition caused by muscular hyperactivity depends highly on selection of the right muscle (s) and the right dose of toxin. Azher and Jankovic [5] were first to note that injection of rectus abdominis (RA) alone could improve posture of some patients with CC. They studied the effect of onabotulinumtoxin A in 9 affected patients. Four of 9 patients improved; one demonstrating 75% improvement of the bent posture. The injected dose of 150–300 units of onabotulinumtoxinA per muscle caused no adverse effects. Todo et al [19] noted that bilateral injection of external oblique muscles (EO) alone with onabotulinum toxin A (75–90 units pe muscle can also significantly improve the CC angle. Wijemanne et al [17] found that initial improvement of CC after bilateral injection of RA can be significantly enhanced by adding bilateral injection of external oblique muscles. Jabbari [20] and de Monte et al [21] injected both rectus abdominis and external oblique muscles with onabotulinumtoxinA and noticed significant improvement of posture and pain in their patient (Table 1). Sole injection of psoas major or iliopsoas muscle with botulinum toxin did not seem to be helpful in improving CC [14,15].

**Figure 1:**
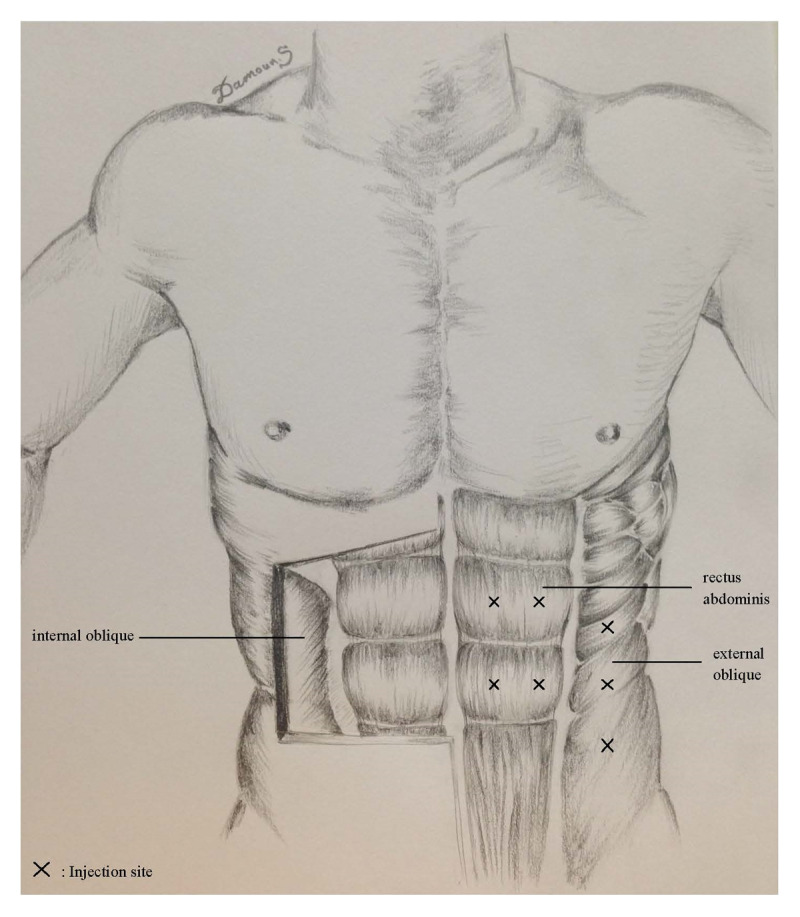
Points of botulinum neurotoxin (botox) injection into the rectus abdominis and external oblique muscles in a 46 years-old, muscular man with several years of severe camptocormia [20]. Injections were bilateral, 40units/point for RA and 30 units/point for EO. Drawing curtesy of Damoun Safarpour, M.D.

The optimum dose of botulinum toxin for treatment of CC would be a dose large enough to produce significant improvement of CC while causing no or minimal side effects. RA and EO are powerful muscles and in most people require a large dose to respond. Although de Monte et al [21] reported satisfaction with a modest total dose of botulinumtoxinA (30units and 50 units per RA and EO, respectively) (Table 1), the authors did not provide information on long term follow up with repeated injections. Jankovic and Azher [5] used 300–600 and Jabbari [20] used 500 units of onabotulinum toxinA per session; both studies reported no side effects. In the latter study, the patient had a 3years follow up with repeated injections every 4 months (Figure 1).

The large doses of botulinum neurotoxins per session used in three studies of this review [5,14,20], is beyond the recommended FDA dose of 400 units per session for onabobotulinum toxinA treatment of spasticity. Recent data, however, has demonstrated that up to 800 units of ona or incobotulinumA can be used safely in one session [22]. However, since injecting high doses of these toxins into abdominal region could potentially cause issues in people with small muscular built, it would be logical to consider the patient’s weight and physical built before considering botulinum toxin therapy for CC. The case reported by Jabbari [20] (Table 1, Figure 1), remained responsive over 3 years of follow up with repeated injections.

Because accurate localization of target muscles is important for both efficacy and safety, therapeutic botulinum neurotoxin administration should ideally be performed with EMG or ultrasound guidance; for deeper targets such as iliopsoas, additional imaging guidance may also be helpful. Only two reported studies performed botulinum neurotoxin injections in camptocormia under ultrasound guidance [16,19].

## Conclusion

A majority of the reports presented in this review suggest efficacy of botulinum toxin injections in improving posture and pain of patients with camptocormia. Confirmation of these positive results, however, requires conduction of double-blind, placebo-controlled investigations. Admittingly, due to rarity of camptocormia, such sizeable studies would be difficult to perform. Furthermore, there is little long-term data since the efficacy of all reported studies is limited. Randomized, blinded studies should carefully select culprit muscles. Based on the limited information from open label studies, combined injection of RA and EO muscles seems to be a reasonable approach. The toxin dose can be based on the patient’s weight but underdosing should be avoided. Furthermore, since several investigators have found that physiotherapy by itself improves comptocormia [23,24,25,26], future studies should include physiotherapy in the BONT treatment protocols of CC.
